# Evaluating E-Visit Utilization and Efficacy for Oral Contraceptive Pills in a Large, Integrated Health Care System

**DOI:** 10.1097/og9.0000000000000121

**Published:** 2025-10-09

**Authors:** Eve Zaritsky, Dana Sax, Emily Bolton, Jeitzel M. Torres-Rodriguez, Ameek K. Bindra, Jie Huang, Mary Reed

**Affiliations:** Obstetrics and Gynecology and Emergency Medicine, Kaiser Permanente Oakland Medical Center, Oakland, and the Division of Research, Kaiser Permanente Oakland Medical Center, Pleasanton, California.

## Abstract

**OBJECTIVE:**

To describe and compare outcomes, including access to care, timeliness of care, and need for a close follow-up visit, by the following visit modalities: office, telephone, video, and e-visit for women seeking oral contraceptive care.

**METHODS:**

We conducted a retrospective cohort study of all Kaiser Permanente Northern California patient-initiated encounters in the department of obstetrics and gynecology with a primary diagnosis related to oral contraceptive care in 2023 among women aged 18–50 years, excluding those who were not pregnant or those seeking nonoral contraceptive methods. Outcomes included the time from care initiation to oral contraceptive order, rates of contraceptive orders and pickups, the time from care initiation to medication pickup, and rates of 7-day in-person clinic follow-up by visit modality. Covariates included age, race and ethnicity, neighborhood socioeconomic status, primary language, and comorbidity burden (Elixhauser Comorbidity Index score). Using multivariable regression, we calculated marginal adjusted estimates by modality.

**RESULTS:**

Among 23,122 encounters, the median patient age was 28 years; 20.9% of the women were Asian, 8.9% Black, 32.7% Hispanic, 36.2% non-Hispanic White, and 1.4% none of the above. Telemedicine was more frequently chosen than in-office visits, with 49.3% choosing visits by telephone, 9.9% by video, and 13.7% by e-visit; 27.1% chose office visits. After adjusting for patient characteristics, women who chose e-visits, compared with those who chose an office visit, were more likely to be Black, Hispanic, or Asian (vs White), more likely to primarily speak English, and less likely to have comorbid illnesses. E-visits had the highest adjusted rate of contraceptive orders (92.0%, 95% CI, 91.1–93.0%) and the lowest adjusted rate of 7-day in-person follow-up. All telemedicine modalities had higher contraceptive pickup rates compared with office visits (61.6%). However, rates were slightly higher for telephone (76.7%) and video (76.2%) visits than for e-visits (73.2%). E-visits were associated with the timeliest care: Contraceptive orders were placed 11.3 days faster (95% CI, 10.9–11.8), and pickups occurred 9.5 days sooner (95% CI, 7.7–11.3) compared with office visits.

**CONCLUSION:**

E-visits for oral contraceptive care may offer a timely and patient-centered method of care delivery with more timely contraceptive orders and pickups and less need for short-term follow-up, compared with other visit modalities. Asynchronous telemedicine presents as an emerging opportunity to increase access to contraceptive care.

## Abstract 2

Asynchronous e-visits for contraceptive care offer a convenient, patient-centered method with timelier orders, faster prescription pickups, less follow-up, and increased reproductive health care access.

Electronic visits (e-visits) are structured, asynchronous communications.^1^ Use of telemedicine, and e-visits in particular, has expanded substantially since the coronavirus disease 2019 (COVID-19) pandemic.^2–7^ Unlike real-time visits, e-visits allow patients to receive care without scheduling an appointment or interacting live with a clinician, making them uniquely suited for straightforward, protocol-driven issues such as contraception. E-visits can offer flexibility by removing the need for simultaneous patient–clinician availability and can improve health care access for those with limited connectivity.^8^

Telemedicine—by telephone and video—has been used effectively for family planning services to expand access to reproductive health services.^9,10^ By the mid-2020s, 84% of surveyed obstetrician–gynecologists had integrated telehealth into their practices, up from 14% prepandemic.^11^ Although e-visits are widely used in primary care,^12–14^ their role in contraceptive access remains underexplored. Yet, improving contraceptive access may reduce maternal mortality and unintended pregnancies.^15^

Understanding patient choices regarding telemedicine, particularly e-visits, is essential for equitable care delivery. Factors such as demographics, clinic access, and digital literacy influence use.^1,16,17^

Kaiser Permanente Northern California (KPNC) launched contraceptive e-visits in 2022. Our study evaluates how this newer modality compares with office, video, and telephone visits in terms of patient characteristics, care timeliness, and follow-up needs.

## METHODS

We conducted a retrospective cohort study using electronic health record data from all female patients aged 18–50 years with active Kaiser Foundation Health Plan memberships for the past 12 months who had any outpatient visit or e-visit in a KPNC obstetrics and gynecology department with a primary diagnosis of contraceptive care between January 1 and December 31, 2023. International Classification of Diseases, Tenth Revision (ICD-10) codes for “contraceptive surveillance,” “contraception management,” “contraceptive method surveillance,” “contraception counseling,” and “contraception initial prescription” were used to identify patients (see Appendix 1, available online at http://links.lww.com/AOG/E356, for codes).^18^ To minimize potential sources of bias, data definitions and coding were consistent systemwide, and all included encounters met uniform inclusion criteria.

We excluded patients with a known pregnancy at the time of their visit and those seeking nonoral contraceptive methods. This is because, within the KPNC e-visit platform, oral contraceptive pills only are available for prescribing through asynchronous care; patients seeking long-acting reversible contraceptives, patches, injections, or other methods are referred to scheduled visits. Additionally, patients seeking only a medication refill (not a new prescription) were excluded from this study, because they use a different, non–e-visit portal tool specifically for refills. Additional exclusion codes can be found in Appendix 1 (http://links.lww.com/AOG/E356). This study was approved by the Kaiser Permanente Institutional Review Board with a waiver for the requirement for written informed consent.

When booking an appointment, patients could choose between scheduling a traditional in-person visit or a telemedicine (telephone or video) appointment, or submitting an asynchronous e-visit, depending on certain screening questions (Appendix 2, available online at http://links.lww.com/AOG/E356). Among eligible visits, we compared patient characteristics such as age, race and ethnicity, language, residential neighborhood socioeconomic status (Census block group level), neighborhood internet access (Census tract level), Elixhauser Comorbidity Index (a validated tool that quantifies a patient's burden of comorbid conditions using ICD-10 diagnosis codes), and history of mobile access across visit types.^19^ We calculated each patient's Elixhauser Comorbidity Index score using ICD-10 codes recorded in the electronic health record system, regardless of visit type; the scoring was uniformly applied across all visit modalities using the same data source.

Because all patients included had active Kaiser Foundation Health Plan memberships, insurance status was uniform across the cohort and was not included as a separate covariate. Regardless of specific insurance plan type within Kaiser Permanente, all members had access to the same visit modalities and prescription options.

To evaluate for potential disparities by race or ethnicity reported in previous literature,^20^ we obtained race and ethnicity data from electronic health records based on patient self-reporting at the time of health plan enrollment or clinical intake. Classification options were defined by the health system and not by the investigators. Categories included Asian, Black, Hispanic, White, and other. Other specifically included American Indian or Alaska Native, Native Hawaiian or other Pacific Islander, patients reporting multiple races or ethnicities, and those with unknown or missing race or ethnicity. We included race and ethnicity as covariates to assess whether these characteristics were associated with study outcomes, including access to different care modalities and timeliness of contraceptive care. The total percentage of missing race or ethnicity data is reported in Table 1 as part of the none of the above category. Racial and ethnic groups are listed alphabetically in all tables.

**Table 1.** Patient Characteristics Among Women With a Women's Health Visit for Contraceptive Care, January 1–December 31, 2023*

Characteristic	All (N=23,122)	Office (n=6,255)	Telephone (n=11,406)	Video (n=2,291)	E-Visit (n=3,170)
Age (y)					
18–25	37.8	38.2	36.1	37.3	43.7
26–30	20.7	20.2	20.6	21.8	21.3
31–35	18.0	17.6	18.3	18.3	17.5
36–50	23.5	24.0	25.0	22.6	17.6
Race and ethnicity					
Asian	20.9	18.2	20.0	27.8	24.4
Black	8.9	9.3	8.3	8.6	10.4
Hispanic	32.7	33.3	34.6	24.3	30.5
White	36.2	38.1	35.6	38.2	33.3
None of the above	1.4	1.2	1.5	1.2	1.4
Language					
English	96.0	94.3	96.2	97.7	97.2
Non-English	3.8	5.6	3.6	2.1	2.1
NDI					
1 (least deprived)	16.4	15.0	15.5	25.5	15.8
2	20.5	20.5	20.4	21.9	19.5
3	23.8	23.9	23.7	22.6	24.5
4	21.8	21.9	22.3	17.4	22.6
5 (most deprived)	17.6	18.7	18.0	12.6	17.5
Neighborhood internet (ACS)^†^					
Less than 90%	19.4	20.1	19.6	15.3	20.4
90–less than 95%	30.1	31.2	29.8	29.3	29.6
95% or more	34.9	34.0	34.7	40.6	33.4
Any comorbidity (Elixhauser Comorbidity Index)					
No	75.1	70.5	75.0	76.3	83.5
Yes	24.9	29.5	25.0	23.7	16.5

NDI, Neighborhood Deprivation Index; ACS, American Community Survey.

Data are column %.

* Percentage of missing data: 0.26% for language, 0.03% for NDI, and 15.57% for neighborhood internet.

† Percentage of household with a broadband internet subscription based on 2018–2022 ACS 5-year estimates.

Additionally, we assessed the rate of oral contraceptive orders linked to the index encounter; the time from care initiation to placement of any contraceptive order (among orders placed); and, among orders picked up, the time from care initiation to contraceptive pickup. *Care initiation* was defined as the date the patient contacted the health system to request contraceptive care—either by submitting an e-visit or calling to schedule a visit—not the date the visit occurred. Finally, we also evaluated rates of in-person gynecology clinic follow-up visits that occurred within 7 days of the index encounter. This 7-day window was selected based on prior telemedicine studies in primary care as a standardized measure to capture short-term return visits, which may indicate potential gaps in initial care delivery or unresolved issues.^21^ Follow-up visits were limited to outpatient obstetrics and gynecology clinic encounters and did not include urgent care or emergency department visits.

Multinomial logistic regression was used to analyze patient characteristics associated with visit type using in-person visits as the reference group. Relative risks (RRs) were calculated by exponentiating the model coefficients, using the rrr option in Stata's mlogit command. Multivariable linear regression was used to evaluate time (in days) between care initiation and both contraceptive order and pickup. Although time variables are typically right-skewed, we conducted sensitivity analyses to assess model robustness, including truncating time variables at the 95th and 99th percentiles and using Poisson regression. These alternative specifications yielded consistent results, supporting the use of linear models for interpretability. Multivariable logistic regression was used to compare binary outcomes such as contraceptive ordering, pickup, and 7-day in-person visits. Standard errors were adjusted for repeated visits by the same patient by clustering observations by the patient with a robust variance estimator. For easier interpretation, we calculated adjusted estimates for each visit type through marginal standardization by using Stata's margins postestimation command. All analyses were conducted using two-sided tests for significance and *P*<.05 as the threshold for significance in Stata 17.0.

## RESULTS

**Fig. 1.**
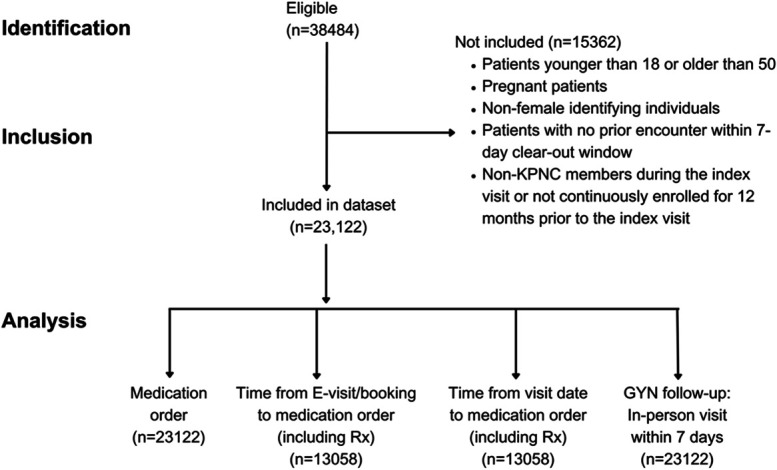
Patient inclusion diagram. KPNC, Kaiser Permanente Northern California.

The study cohort included 23,122 patients with visits for contraception in 2023 (Fig. 1). The median age was 28 years; 20.9% of patients were Asian, 8.9% were Black, 32.7% were Hispanic, 36.2% were non-Hispanic White, and 1.4% were none of the above, which included American Indian or Alaska Native; Native Hawaiian or other Pacific Islander; and multiple races or ethnicities, unknown, or missing (Table 1). We found that 17.6% of patients lived in the most deprived neighborhoods, and 34.9% of patients resided in neighborhoods with at least 95% household broadband internet access. Most patients had no comorbidities (75.1%). Telemedicine was more frequently chosen than in-office visits (n=6,255, 27.1%) and included e-visits (n=3,170, 13.7%), telephone visits (n=11,406, 49.3%), and video visits (n=2,291, 9.9%).

*Zaritsky. E-Visit Utilization and Efficacy for OCPs. O&G Open 2025*.

Table 2 shows differences in patient characteristics by visit modality using office visits as the comparator, with differences displayed as RR. Patients aged 36–50 years (vs those aged 18–25 years) were significantly less likely to choose an e-visit over an office visit (RR 0.7, 95% CI, 0.6–0.8). Women older than age 25 years were more likely than those aged 18–25 years to choose a telephone visit over an office visit (26–30 years: RR, 1.1; 95% CI, 1.0–1.20; 31–35 years: RR, 1.1: 95% CI, 1.0–1.3; 36–50 years: RR, 1.2; 95% CI, 1.1–1.3). Compared with White patients, Asian, Black, and Hispanic patients were significantly more likely to choose an e-visit over an office visit, with RRs of 1.6 (95% CI, 1.4–1.8) for Asian patients, 1.4 (95% CI, 1.2–1.6) for Black patients, and 1.1 (95% CI, 1.0–1.3) for Hispanic patients. Hispanic and Asian patients also were significantly more likely to choose telephone visits over office visits, with RRs of 1.2 for both groups (95% CI, 1.1–1.3 for both Asian and Hispanic patients). Asian patients were significantly more likely to choose video than office visits (RR, 1.6; 95% CI, 1.4–1.8). English-speaking patients were significantly more likely to select any telemedicine visit compared with an office visit; telemedicine visits included e-visits (RR, 2.7; 95% CI, 2.1–3.6), telephone (RR, 1.8; 95% CI, 1.5–2.0), and video (RR, 2.6; 95% CI, 1.9–3.5). Neighborhood socioeconomic status (Neighborhood Deprivation Index) was not significantly statistically associated with visit choice other than for video visits, in which patients living in more impoverished neighborhoods were significantly less likely to choose video visits over office visits. Patients with comorbidities were significantly less likely to use any telemedicine modality compared with those without comorbidities, with the greatest difference observed for e-visits (RR, 0.5; 95% CI, 0.4–0.5), followed by telephone (RR, 0.8; 95% CI, 0.7–0.8), and video (RR, 0.8; 95% CI, 0.7–0.9). There were no statistically significant differences in visit type based on neighborhood internet access.

**Table 2.** Patient Characteristics Associated With Telemedicine Visit Type (E-Visit, Telephone, or Video)*

Characteristic	E-Visit	Telephone	Video
Age (y)			
18–25 (ref)			
26–30	1.0 (0.9–1.1)	1.1 (1.0–1.2)	1.1 (1.0–1.3)
31–35	0.9 (0.8–1.1)	1.1 (1.0–1.3)	1.1 (1.0–1.3)
36–50	0.7 (0.6–0.8)	1.2 (1.1–1.3)	1.0 (0.8–1.1)
Race and ethnicity			
Asian	1.6 (1.4–1.8)	1.2 (1.1–1.3)	1.6 (1.4–1.8)
Black	1.4 (1.2–1.6)	1.0 (0.9–1.1)	1.1 (0.9–1.3)
Hispanic	1.1 (1.0–1.3)	1.2 (1.1–1.3)	0.9 (0.8–1.0)
White (ref)			
None of the above	1.3 (0.9–2.0)	1.3 (1.0–1.8)	1.1 (0.7–1.7)
Language			
English	2.7 (2.1–3.6)	1.8 (1.5–2.0)	2.6 (1.9–3.5)
Non-English (ref)			
NDI			
1 (least deprived, ref)			
2	0.9 (0.8–1.1)	1.0 (0.9–1.1)	0.7 (0.6–0.8)
3	1.0 (0.9–1.2)	1.0 (0.9–1.1)	0.6 (0.5–0.7)
4	1.0 (0.9–1.2)	1.0 (0.9–1.1)	0.5 (0.5–0.6)
5 (most deprived)	0.9 (0.8–1.1)	1.0 (0.9–1.1)	0.5 (0.4–0.6)
Any comorbidity			
No (ref)			
Yes	0.5 (0.4–0.5)	0.8 (0.7–0.8)	0.8 (0.7–0.9)
Neighborhood internet			
Less than 90% (ref)			
90–less than 95%	0.9 (0.8–1.0)	1.0 (0.9–1.1)	1.0 (0.9–1.2)
95% or more	0.9 (0.8–1.0)	1.0 (0.9–1.1)	1.1 (0.9–1.3)

NDI, Neighborhood Deprivation Index.

Data are adjusted relative risk (95% CI).

* Model: multinomial logistic regression with office visit as the reference. Standard errors are adjusted for visits by the same patient.

**Fig. 2.**
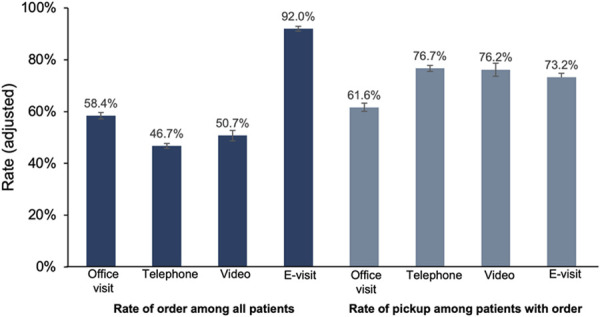
Adjusted percentage of contraceptive orders and pharmacy pickups by visit type.

After adjusting for all covariates listed in Table 1, e-visits had the highest rate of contraceptive orders, with 92.0% (95% CI, 91.1–93.0%) of e-visits resulting in a contraceptive order. In contrast, office (58.4%; 95% CI, 57.2–59.6%), telephone (46.7%; 95% CI, 45.8–47.7%), and video visits (50.7%; 95% CI, 48.7–52.8%) had lower rates of contraceptive orders. All telemedicine modalities had higher contraceptive pickup rates in the pharmacy (or delivered by mail): 73.2% (95% CI, 71.6–74.8%) for e-visits, 76.7% (95% CI, 75.6–77.8%) for telephone visits, and 76.2% (95% CI, 73.7–78.7%) for video encounters, compared with 61.6% (95% CI, 60.0–63.2%) for office visits (Fig. 2, Appendix 3, available online at http://links.lww.com/AOG/E356).

*Zaritsky. E-Visit Utilization and Efficacy for OCPs. O&G Open 2025*.

**Fig. 3.**
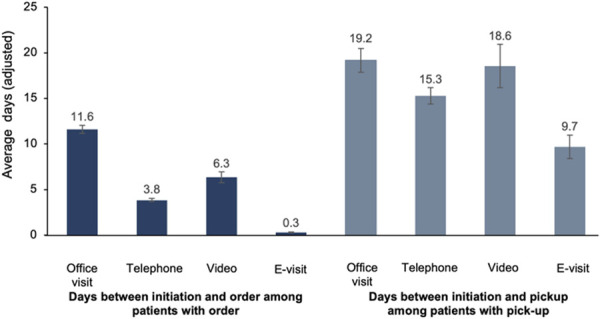
Adjusted days between care initiation and contraceptive order and care initiation and contraceptive pickup by visit type.

After adjusting for patient characteristics, we found e-visits had the shortest interval between care initiation and contraceptive order among all visit types, followed by telephone, video, and office visits. The mean time from care initiation to medication order was 11.3 days (95% CI, 10.9–11.8) longer for office visits compared with e-visits (Fig. 3). Among visits in which the patient picked up the contraceptive (or had it filled for mail delivery), the time from care initiation was again shortest for e-visits, with the mean time from contraceptive order to pickup 9.5 (95% CI, 7.7–11.3) days longer for office visits compared with e-visits. Compared with e-visits, the difference in days between the visit and contraceptive pickup was shorter for office encounters for asynchronous visits, 1.8 days (95% CI, 0.05–3.50 days) longer for telephone visits, and 3.0 days (95% CI, 0.5–5.6) longer for video visits (Appendix 4, available online at http://links.lww.com/AOG/E356).

*Zaritsky. E-Visit Utilization and Efficacy for OCPs. O&G Open 2025*.

**Fig. 4.**
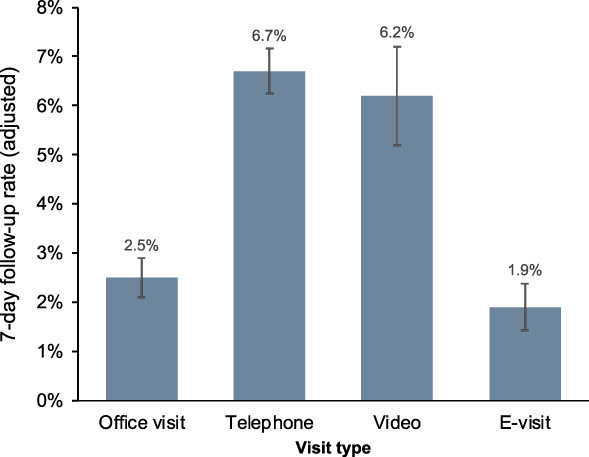
Adjusted rates of 7-day in person obstetrics and gynecology follow-up by visit type.

Figure 4 shows that overall, rates of in-person obstetrics and gynecology follow-ups within 7 days of the index visit were low across all visit modalities, although telephone (6.7%; 95% CI, 6.3–7.2%) and video (6.2%; 95% CI, 5.2–7.2%) encounters had significantly higher rates compared with e-visits (Appendix 5, available online at http://links.lww.com/AOG/E356).

*Zaritsky. E-Visit Utilization and Efficacy for OCPs. O&G Open 2025*.

## DISCUSSION

In a large, diverse, multicenter population of women seeking contraceptive care in an integrated health delivery system in 2023, our findings suggest that telemedicine options such as telephone, video, and e-visits offer greater choice and more timely access. E-visits had the shortest time from care initiation to contraceptive order and pickup and were associated with the lowest rates of in-clinic follow-up within 7 days.

Our findings align with existing research in primary care and women's health showing that telemedicine reduces wait times, improves access, and enhances efficiency.^22–26^ Studies by Graetz et al and Hong et al found that telemedicine—particularly video—was associated with faster appointment completion and more prescribing activity, with lower follow-up needs.^2,27^ Contraceptive care is often transactional, with patients seeking a specific outcome. Our findings suggest that e-visits can effectively fulfill this goal.

Although telemedicine is efficient, some patients may have concerns. During the COVID-19 pandemic, a national survey found that only 17% of respondents who received contraceptive services used telehealth and rated clinicians lower on respect, shared decision-making, and preference consideration.^28^ These results suggest opportunities to improve patient-centered care across telemedicine modalities. Further research should explore how visit modality affects satisfaction and preferences, particularly among patients with greater complexity or risk. Additionally, although e-visits offer efficient contraceptive access within KPNC, the recent approval of the progestin-only norgestrel (0.075 mg) for over-the-counter use has the potential to further expand access to contraception.

Telemedicine also may expand access to reproductive care across diverse populations. We found that Black, Hispanic, and Asian patients were significantly more likely to choose an e-visit over an office visit; Hispanic and Asian patients were significantly more likely to choose a telephone over an office visit; and Asian patients were significantly more likely to choose a video visit over an office visit. Although the specific reasons for these differences are not clear from study findings, this may be related to patients' concerns about discrimination or bias when seeking in-person care or that factors such as transportation, time off work, childcare, or language barriers affect patient choice of visit modality. Future studies should examine these drivers and support equitable care delivery across modalities.

The main limitation is the retrospective study design, which may limit capture of patient-level differences across visit types or introduce misclassification. There are likely selection biases in visit choice that we were unable to measure by using available electronic databases. These differences may have affected their likelihood of receiving a contraceptive order and how quickly they were able to pick up medication. Women who choose e-visits may be more confident in their contraceptive decision, have less health comorbidities, and, therefore, be more likely to have an order placed and medication picked up. It is possible that women who seek in-person care may have had more complex needs beyond contraception and this may have contributed to the lower contraceptive order rate in this group.

In addition, we did not assess the efficacy of the contraceptive start, because only prescription pickup was measured; we do not know how long the contraceptive was continued or how satisfied the patient was with the contraceptive method choice. Although we used the primary diagnosis codes for patient inclusion, miscoding may have occurred, and this may have not been evenly distributed across visit modalities. Additionally, our findings may have limited external generalizability to health care systems with more limited access to telemedicine or different reimbursement structures. Other institutions may have different electronic health record platforms and clinical workflows that could affect the implementation and effectiveness of telemedicine interventions.

We only focused on prescriptions for oral contraceptives because these are the most common contraceptive modality within KPNC and the only method available from e-visits.^29^ Further research should more broadly examine all contraceptive methods across different visit types. Additional studies also should explore patient preferences in visit types to enable physicians to offer e-visits more effectively, particularly to groups that may prefer this option.

Strengths of our study include a large, diverse, multicenter, community-based population with a large sample size, allowing for a broad assessment of contraceptive access and timeliness of care across sites and populations and adjustment for multiple patient and clinician characteristics. Our findings highlight the potential benefit of e-visits, which may improve access to contraceptive care in a diverse, community-based setting, with potential generalizability to other settings and populations.
